# First Report of Seropositivity to *Trypanosoma cruzi* in Mexican Afro-Descendants from Guerrero and Oaxaca States

**DOI:** 10.1155/2024/2014142

**Published:** 2024-05-08

**Authors:** Bertha Espinoza, Hilda Rangel-Flores, Gabriel Saucedo-Arteaga, Ignacio Martínez, Carlos A. Aguilar-Salinas, Brenda Cabrera-Mendoza, David Ponce-Patiño, Javier Mendoza-Duarter, Carlos Eduardo Martínez-Rangel

**Affiliations:** ^1^Departamento de Inmunología, Instituto de Investigaciones Biomédicas, UNAM, Ciudad de México 04010, Mexico; ^2^Centro de Investigación Sobre Enfermedades Infecciosas, Instituto Nacional de Salud Pública (INSP), Cuernavaca, Morelos 62100, Mexico; ^3^Instituto Nacional de Ciencias Médicas y Nutrición Salvador Zubirán (INCMNSZ), Ciudad de México 14080, Mexico; ^4^Hospital Regional de Alta Especialidad, Centenario de la Revolución Emiliano Zapata, ISSSTE, Emiliano Zapata, Morelos 62765, Mexico; ^5^Facultad de Medicina, Universidad Autónoma del Estado de Morelos, Cuernavaca, Morelos 62209, Mexico

## Abstract

Mexican Afro-descendant is a population poorly studied in many aspects, between them the infectious diseases that they suffer. This population is mainly found in the country's Pacific (Oaxaca and Guerrero states) and Atlantic (Veracruz) coast. In these regions, a diversity of triatomine vectors of the Chagas disease is found. Also, all the genotypes of *Trypanosoma cruzi* DTUs have been reported. That is why the present study aimed to study the presence of antibodies against *T. cruzi* and cardiac pathology associated with the Chagas disease in the Mexican Afro-descendant population of Guerrero and Oaxaca. ELISA, Western blot, and recombinant antigen's ELISA were used to evaluate the seropositivity of these communities. Furthermore, an electrocardiographic study and evaluation of risk factors associated with *T. cruzi* infection in the Oaxaca and Guerrero populations were conducted. 26.77% of the analyzed population was positive for two serological tests. These percentages are higher than the previously reported for the mestizo population in similar studies. Electrocardiographic results showed cardiac disorder associated with the Chagas disease in the population. Also, risk factors were identified associated with the men's activities in the outdoor working areas.

## 1. Introduction

The Chagas disease (CD) is caused by the protozoa *Trypanosoma cruzi*, a parasite transmitted by vectors of the subfamily Triatominae (Hemiptera: Reduviidae). They infect when defecating on the skin or in humid membranes. Although vector transmission is the most common form of getting infected, there are other ways of transmission of the parasite, including oral ingestion of contaminated food and liquids, blood transfusion and organ transplants, mother-to-child transmission, and laboratory accidents. The CD is one of the leading health problems provoked by parasites in Latin America, where 21 countries are endemic to the disease. It is estimated that 6 to 7 million people are infected worldwide, mostly in Latin America [1].

Mexico is a CD-endemic country. In Mexico, previous reports indicate that in the 2007-2016 period, Guerrero and Oaxaca states (both on the Mexican Pacific coast), as well as Veracruz (on the Mexican Atlantic Coast), were states with the highest incidence of CD [2, 3]. In addition, several species of vectors infected with *T. cruzi* have been reported in these states [4].

In Mexico, the Afro-descendant communities have a significant presence in the states of Veracruz, Guerrero, and Oaxaca, where socioeconomic conditions bring them closer to poverty [5]. This condition favors that almost a third of Afro-descendants from 6 to 17 years old may suffer some degree of malnutrition [6, 7]. This, in turn, makes them more vulnerable to having poor or inadequate health compared to other Mexican groups [8, 9]. Despite these facts, the occurrence of CD in these specific groups has not been studied.

To know the seroprevalence of *T. cruzi* in this population is essential since studies carried out in Afro-descendant communities in other countries, such as Brazil, have reported contradictory results. Some authors have suggested that infected Afro-descendants, particularly those over 80 years old, have a greater probability of developing severe electrocardiographic abnormalities associated with CD and propensity to die [10, 11]. At the same time, other studies indicate that some communities where Afro-descendants know the vector and have reported contact with it have little seropositivity to *T. cruzi* [12].

In Mexico, the presence of *T. cruzi* infection and CD-associated pathology is unknown in this population. Therefore, the aim of this study was to establish the seropositivity to *T. cruzi* in Mexican Afro-descendants from some communities in Oaxaca and Guerrero. Additionally, an electrocardiogram was done on those seropositive individuals, and a study was conducted to identify risk factors associated with this condition in these populations.

## 2. Materials and Methods

### 2.1. Localities Studied and Participants

**Figure 1:**
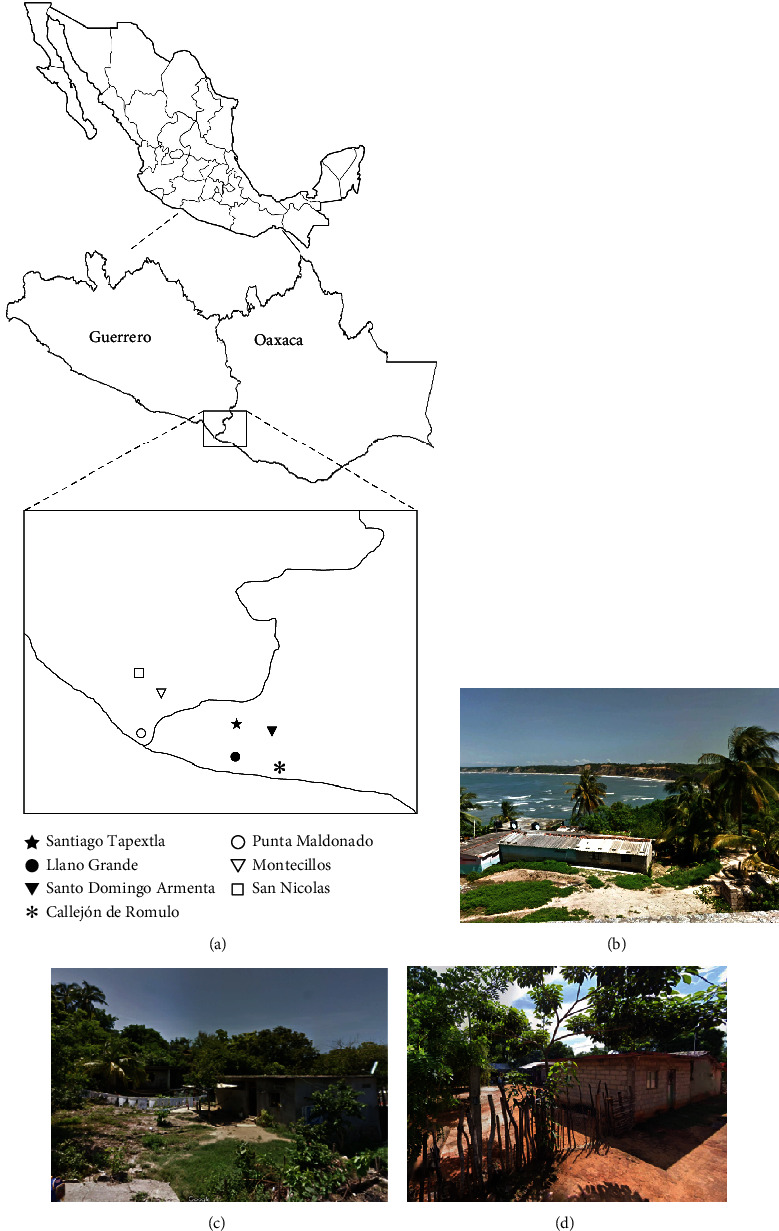
Location and names of the communities evaluated on the Pacific coast. (a) Geographic location of the communities studied in Guerrero and Oaxaca. Representative images of the houses in the communities of (b, c) Punta Maldonado and (d) Montecillos in the state of Guerrero.

The samples were collected in several localities on the Mexican Pacific coast (Oaxaca and Guerrero states) where Afro-descendants live. Four locations were visited in the Oaxaca State: Santiago Tapextla (16° 19′ 34.1^″^ N, 98° 34′ 00.6^″^ W), Llano Grande (16° 18′ 11.4^″^ N, 98° 26′ 37.3^″^ W), Santo Domingo de Armenta (16° 19′ 53.4^″^ N, 98° 22′ 41.5^″^ W), and Callejon de Romulo (16° 16′ 24.9^″^ N, 98° 21′ 10.0^″^ W). In the state of Guerrero, 3 locations were visited: Punta Maldonado (16° 19′ 34.1^″^ N, 98° 34′ 00.6^″^ W), Montecillos (16° 23′ 08.6^″^ N, 98° 30′ 12.5^″^ W), and San Nicolas (16° 24′ 40.3^″^ N, 98° 30′ 53.1^″^ W) (Figure 1).

These locations were chosen because the families define themselves as Afro-descendant and recognized as such by public institutions and neighboring communities. All members of the communities, men or women over 18 years old, were invited to participate voluntarily and through informed consent. The exclusion criteria were pregnancy, acute infections, dehydration, or emotional crisis at the time of the study. The Ethics Committee approved the protocol for Human Research of the INCMNSZ (CIIBH: EDN-391-11-14-1), according to the WMA Declaration of Helsinki (2017).

A peripheral venous blood sample was taken from the participants after fasting for at least 8 hours. The puncture was performed by trained personnel in a medical unit, following the procedures established in the Mexican Official Standard NOM-012-SSA3-2012. The sample was centrifuged in situ (3 min × 10^3^ rpm) to separate the serum. The samples were kept at 4°C for transport and aliquoted and frizzed until use.

### 2.2. Serological Determination of Antibodies against *T. cruzi*

An extract of *T. cruzi* epimastigotes Queretaro strain (TBAR/MX/0000/Queretaro) was prepared by sonication as previously described [13]. As reported previously, an in-house immunoenzymatic assay (ELISA) was performed with *T. cruzi* antigen [14]. The cutoff value (COV) was set using the optical density (OD) average from negative controls plus 2.5 standard deviations. A sample was considered positive if their OD/COV division quotient was equal to or greater than 1.

Western blot. *T. cruzi* epimastigote antigen, prepared as has been previously described, was used for the Western blot assays [13]. The recognition of at least one antigen-specific band in the evaluated serum sample was considered a positive result since the negative controls did not present any specific signal [15]. The antigens recognized by each serum were identified by comparing the bands found against a molecular weight marker (26619, Thermo) as previously described [15]. The established values were indicated in kDa.

A third assay was used for serum samples with inconclusive results: the BioElisa Chagas kit (Biokit, Spain) following the supplier's instructions. The samples were evaluated in duplicate, and the absorbance was read in an iMark microplate reader (Bio-Rad, USA). The samples were established as positive or negative according to the supplier's criteria.

### 2.3. Electrographic Study

All the participants with two serological positive results had an electrographic study. Twice as many seronegative individuals were invited to participate in the electrocardiographic evaluation as controls. The study's objective and the procedure for electrocardiographic recording were explained to all participants. Their informed consent was obtained, and the study was carried out by placing the electrodes to obtain the bipolar DII derivative at rest using a portable electrocardiograph. All recording tests were performed and interpreted by qualified medical personnel following the classification of electrocardiograms and an abbreviated referral system for population surveys concerning CD established previously [16].

### 2.4. Epidemiological Data Analysis

A fifty-question questionnaire was applied to the participants. The questions were grouped into several categories: personal data, pathological and nonpathological antecedents, high-risk practices, and obstetrical antecedents (for women). To determine if some variables were correlated with the seropositive results of the Mexican Afro-decedent population, odds ratios (OR) were calculated using online MedCalc Statistical Software 2017 () with 95% confidence intervals. A variable indicates that the exposure is associated with higher odds of the outcome if OR > 1. An OR < 1 indicates that the exposure is associated with a lower odd of the outcome [17].

The Pearson chi-square test was performed to establish whether there was a statistically significant association between the positive serological result and the geographical origin, age, and sex of the participants, as previously described [18]. The online platform Social Science Statistics () was used for this.

## 3. Results

### 3.1. House Conditions

Generally, the Mexican Afro-descendant community in the southern states lives in rustic households with zinc, asbestos, or wood roofs; the floors are made of cement, wood, or earth (Figure 1) [7].

### 3.2. Serological Findings

**Figure 2:**
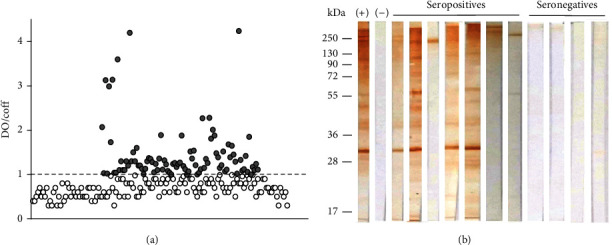
ELISA and WB of sera from participants. (a) ELISA results, the assay was performed as described in Materials and Methods. Seronegative (white circles) and seropositive (grey circles) samples are shown. The optical density/cutoff point ratio was used in the graph. The dotted line represents the cutoff value. (b) Western blot showing antigens recognized by seropositive sera. Seropositive serum was used as positive control (+); negative serum from a healthy individual was used as negative control (-). Some sera from seronegative participants are included. Representative positive and negative sera are shown.

The study consisted of 239 participants from Oaxaca and Guerrero. Of them, 91 were positive to the ELISA assay, and 35 were confirmed as positive by Western blot. Of those WB negative, 29 were confirmed as positive by the Bioelisa Chagas assay, for a total of 64 seropositive with two positive tests, representing a seropositivity of 26.77%. Diversity was found in the serological response evaluated by ELISA since the dispersion of the data shows that some samples had high OD values. In contrast, others barely exceeded the cutoff point (Figure 2(a)).

The Western blot results show heterogeneity in the antigens recognized by the positive individuals. The positive sera recognize various antigens, some with greater intensity than others. Some sera only recognize one antigen preferentially, while others recognize multiple *T. cruzi* antigens (Figure 2(b)). A more detailed analysis indicates that few antigens were recognized by more than 10% of the evaluated sera. More than half of the sera recognized a group of antigens of >250 kDa (Table 1).

### 3.3. Electrocardiographic Findings

A sample of 61 participants with positive ELISA and WB agreed to participate in the electrocardiographic evaluation. Six of them (9.8%) showed electrocardiographic changes such as myocardial infarction, ischemic heart disease, and complete or incomplete left or right bundle branch block (BBBH). Finally, 64 participants with a negative serological result agreed to participate, and 4 of them (6.25%) showed alterations such as ischemic heart disease, sinus tachycardia, or RIBBBH. The participants with one positive ELISA and WB serology who presented heart disease were all over 50 years old, while the heart patients with two positive ELISA and negative WB serology were all under 45 years old. Seronegative participants with heart disease have a wide age range, and only one individual has RIBBBH, a symptom associated with CD.

On the other hand, based on the data collected in the *ad hoc* questionnaires, an analysis was performed to determine the risk factors among seropositive participants. From this study's 24 epidemiological characteristic analysis, only a few were associated with seropositive to *T. cruzi*. Odds ratios and confidence intervals showed that to have been born in Oaxaca and to have ocular lesions (probable Romaña sign) were associated with being positive for *T. cruzi*. Meanwhile, being men from the Montecillos community and knowing the vector were significant risk factors in Guerrero (Table 2).

## 4. Discussion

Scarce information exists about Mexican Afro-descendants, and it is difficult to know their origin due to the prohibition of trading enslaved people by the Spanish government in the XVI century. The origin of the Afro-descendant population in Mexico is unknown since there are no records on the identity and geographical origin of these people [19]. Currently, 2% of the Mexican population recognizes themselves as African descendants [20]. 40% of them are between 30 and 59 years old. The original African population was forced to migrate to different parts of the country. Nowadays, they are present in all the Mexican territory, which concentrated in four states: Oaxaca, Guerrero, Veracruz, and Estado de México.

In these states, a percentage of Afro-descendants suffer from the same health problem as many Mexicans and 17.7% of the total population of the Afro-descendant community does not have affiliation with any health service. The children who belong to the community have some characteristics of health problems; one child in four has some degree of malnutrition; one in every five children is overweight or obese; children in this community have almost a 40% height deficit [5, 7]. Afro-descendant adults have chronic health problems, where diabetes (13.2%), high blood pressure (32.7%), obesity (74.4% in women and 88.9% in men), and cancer stand out [6]. The leading causes of death are breast and prostate cancer, heart disease, diabetes, liver cirrhosis, high blood pressure, and physical violence [7]. Furthermore, their housing conditions facilitate the coexistence of people with vectors and enable infection with *T. cruzi*.

Mexico is one of the countries with the most vector species for *T. cruzi* transmission, with more than 30 species of triatomine insects. Several species of these insects have been reported in the states of Oaxaca and Guerrero, which are of medical importance because they are infected with *T. cruzi*. Also, in these regions, infected human cases with cardiac symptoms have been reported [15].

In epidemiological terms, few studies in Mexico associate the presence of infectious pathogens in Afro-descendant populations. Some works in other Latin American countries have reported the prevalence of viral [21], bacterial [22], and protozoan [23] infections, particularly in Colombia and Brazil.

The study of infection with *T. cruzi* in Afro-descendants has been addressed mainly in Brazil. Some works have found low seropositivity to *T. cruzi* in Afro-descendant populations, attributed to improvements in housing conditions [12]. Others have suggested that in some regions of the Americas, being an Afro-descendant man, over 80 years old, and infected with *T. cruzi* are factors that predispose mortality [11]. On the other hand, studies have reported that Black or Afro-descendant people are less susceptible to the adverse effects of treatment with benznidazole, which allows them to adhere more successfully to treatment [24].

Our results show that the total prevalence of anti-*T. cruzi* antibodies for the Afro-descendant population analyzed is 26.77% in Oaxaca/Guerrero communities. This percentage is higher than the prevalence reported in works with similar numbers of participants in Brazil [12]. The seropositivity data in this population are also higher than those reported by other works in which the open blood donor population is evaluated in the Pacific Mexican coasts [25]. Preliminary data in our laboratory indicate that a similar seropositivity percentage could be found in some Afro-descendant communities in the state of Veracruz on the Atlantic coast of the country (data not shown).

Interestingly, sera from seropositive participants recognized different antigens than sera from central Mexico seropositive individuals, as previously reported [15]. In addition, the population analyzed in this work had a low recognition of the 25 kDa antigen, which could correspond to that of the 23-28 kDa antigen, reported by our group, as one of the most immunogenic in the mestizo Mexican population [15]. Other groups have also reported this antigen as one of the most identified by Western blot in other populations not identified as Afro-descendants [26].

This difference between the antigens recognized by the sera from Oaxaca-Guerrero Afro-descendant and central Mexico seropositive population could have its origin in the *T. cruzi* strains circulating in each region. Even though it has only been documented the presence of TcI in Oaxaca-Guerrero and central Mexico [27, 28], the presence of TcI subgroups in Mexico has yet to be explored, as it has been recognized in other regions of America [29, 30]. This difference could also be due to the diversity of the transmitting vectors in each region since some species are present in Oaxaca-Guerrero (*T. mazzotti*, *T. picturata*, *T. rubida*, and *T. phyllosoma*) or central Mexico (*T. (Meccus) pallidipennis*, *T. mexicana*, and *T. barberi*) [2, 31]. The differences in antigenic recognition could also be due to the immune response of the Mexican Afro-descendant population, which should be explored in the future.

Some works have proposed that the changes in the electrocardiograms are more evident in Black people infected with *T. cruzi* than in White people, which could be associated with their precarious living and feeding conditions [10].

In the present work of the 6 seropositive participants with electrocardiographic alterations, three of them presented RBBBH, which is consistent with the Mexican mestizo population [15], one of them was diagnosed with BRIHH, and one more with ischemic heart disease.

We also found seronegative participants with heart disease whose etiology is unknown. Statistically, there is no association between infection with *T. cruzi* and the presence of heart disease in the population studied. However, a paired study is still pending to establish whether the presence of heart disease in Mexican Afro-descendants is more frequent than in the mestizo Mexican population.

Regarding the data analyzed, we found that being a man born in the state of Oaxaca and knowing the transmitting vector significantly increases the risk of being seropositive to *T. cruzi*. Because, in the studied communities, men work in the fields and sometimes have to sleep away from home to take care of the crops, which would favor their contact with wild vectors. It is striking that something similar was not found for the inhabitants of Guerrero, whose geographical and social situation is very similar. This should be evaluated in greater detail to find the causes of this result.

This initial study on the presence of CD in some Mexican Afro-descendant populations must motivate more studies about this and other infections that affect these communities that are living in deprived conditions and need more government health facilities. It will be important in the future that these kinds of studies and other genetic, environmental, and lifestyle studies will be considered for developing precision medicine that will translate the data on the medical status of the Mexican Afro-descendant into appropriate healthcare systems as has been proposed for other Black and Afro-descendant populations [32].

## 5. Conclusions

The Mexican Afro-descendant is a population living on the country's Pacific coast, where the ecological and social conditions (vectors, weather, and poverty) determine the infection with *T. cruzi*. This work found 26.77% of seropositivity, a percentage higher than the mestizo population. Cardiopathies associated with Chagas diseases were found, and some risk factors were identified. More studies should be done in this population to develop public health initiatives to improve diagnostic and clinical studies.

## Figures and Tables

**Table 1:** Antigens of *T. cruzi* recognized by seropositive individuals.

Protein (kDa)	Recognition (%)
<17	8.6
17	5.7
25	5.7
27	5.7
30	2.8
32	14.2
34	5.7
37	5.7
40	8.6
43	14.2
50	8.6
54	2.8
59	5.7
63	2.8
70	2.8
75	2.8
79	2.8
82	2.8
87	11.4
91	5.7
95	5.7
104	2.8
109	5.7
115	11.4
140	5.7
149	5.7
152	8.6
163	11.4
200	2.8
230	5.7
250	8.6
>250	51.4

**Table 2:** Risk factors in Oaxaca-Guerrero Mexican Afro-descendant population.

Factor	*T. cruzi* (-)	*T. cruzi* (+)	Odds ratio	CI (95%)	*p* value
Know the vector (Guerrero)					
Yes	25	8	1.36	0.4492–4.1173	0.5864
No	34	8			
Know the vector (Oaxaca)					
Yes	20	6	0.675	0.1963–2.3216	0.5329
No	18	8			
Know the vector (Montecillos)					
Yes	7	6	12.86	1.2899–128.1496	0.0295
No	15	1			
Have you seen the vector in your community? (Oaxaca)					
Yes	15	3	0.4222	0.0969-1.8393	0.2508
No	19	9			
Have you seen the vector in your community? (Guerrero)					
Yes	17	3	0.5701	0.1440-2.2573	0.4235
No	42	13			
Have had blood transfused (Oaxaca)					
Yes	3	2	1.7222	0.2252–11.6221	0.5768
No	31	12			
Have had blood transfused (Guerrero)					
Yes	4	1	0.85	0.0882–8.1923	0.8882
No	51	15			
You have dogs at home (Oaxaca)					
Yes	28	14	10.6842	0.5840-195.4781	0.1102
No	10	0			
You have dogs at home (Guerrero)					
Yes	45	13	1.3481	0.3353-5.4198	0.6739
No	14	3			
Rustic house (Oaxaca)					
Yes	22	6	0.7273	0.1978-2.6740	0.6317
No	16	6			
Rustic house (Guerrero)					
Yes	30	6	0.58	0.1867-1.8019	0.3463
No	29	10			
Born in Guerrero					
Yes	55	10	0.3727	0.1577-0.8809	0.0245
No	41	20			
Born in Oaxaca					
Yes	39	19	2.5245	1.0823-5.8884	0.0321
No	57	11			
Has drank raw milk (Oaxaca)					
Yes	22	5	0.4545	0.1252-1.6507	0.2308
No	16	8			
Has drank raw milk (Guerrero)					
Yes	34	8	0.8403	0.2692-2.6230	0.7645
No	25	7			
Mother was born in Oaxaca					
Yes	24	8	0.6667	0.1346-3.3031	0.6195
No	6	3			
Mother was born in Guerrero					
Yes	19	9	3.2211	0.9425-11.0085	0.0621
No	34	5			
Palpitations					
Yes	9	4	1.2889	0.3243-5.1222	0.7185
No	29	10			
Chest pain					
Yes	16	4	0.5250	0.1389-1.9841	0.3421
No	21	10			
Gender female (Oaxaca)					
Yes	29	11	1.7069	0.3175-9.1774	0.5333
No	9	2			
Gender female (Guerrero)					
Yes	41	8	1.1707	0.2779-4.9314	0.8299
No	18	3			
Gender male (Oaxaca)					
Yes	9	2	0.6591	0.1207-3.6001	0.6303
No	29	11			
Gender male (Guerrero)					
Yes	18	3	0.8542	0.2028-3.5980	0.8299
No	41	8			
Gender male (Montecillos)					
Yes	2	4	9.5000	1.2717-70.9666	0.0282
No	19	4			
Romaña sign (Oaxaca)					
Yes	1	3	11.6667	1.0810-125.9073	0.0429
No	35	9			

## Data Availability

The results will be available under request to the corresponding author.
